# Acute Inferior Wall Myocardial Infarction due to Occlusion of the Wrapped Left Anterior Descending Coronary Artery

**DOI:** 10.1155/2013/983943

**Published:** 2013-07-24

**Authors:** Thottuvelil Narayanan Sunil Roy, Jafar Saeed Nagham, Rajappan Anil Kumar

**Affiliations:** Department of Cardiology, Belhoul Speciality Hospital, P.O. Box 5527, Dubai, UAE

## Abstract

Acute occlusion of the left anterior descending coronary artery (LAD) generally results in ST segment elevations in precordial leads and reciprocal ST segment depression in inferior leads. The occurrence of isolated inferior myocardial infarction due to occlusion of LAD is very rare. We describe an isolated acute inferior myocardial infarction due to occlusion of a wrapped LAD at the apex which continues as the large posterior descending coronary artery (PDA) beyond the occlusion.

## 1. Introduction

Acute occlusion of the left anterior descending coronary artery (LAD) generally results in ST segment elevations in precordial leads and reciprocal ST segment depression in inferior leads. When ST segment elevation occurs in inferior leads, the culprit artery is either the right coronary artery (RCA) or left circumflex coronary artery (LCX). Simultaneous anterior and inferior myocardial infarction has been described due to occlusion of “wrapped LAD” [1]. The occurrence of isolated inferior myocardial infarction due to occlusion of LAD is unknown. Here we describe an isolated acute inferior myocardial infarction due to occlusion of LAD which continues as the posterior descending coronary artery (PDA).

## 2. Case Report

**Figure 1:**
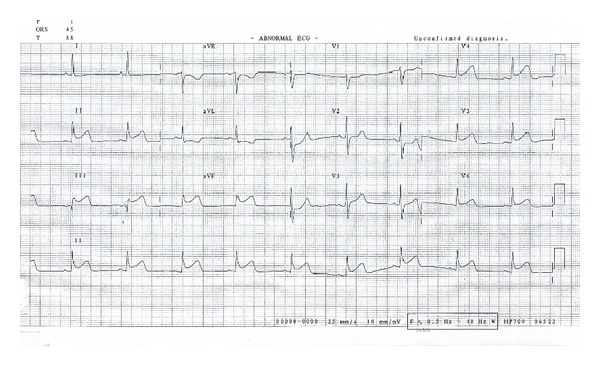
12-lead ECG showing hyperacute phase of inferior wall myocardial infarction. ST segment elevation is seen in leads II, III, and AVF with upright T wave merging with the ST segment. ST elevation is also noticed in V5 and V6.

A 41-year-old previously healthy male presented to our emergency department with severe crushing chest pain of 2-hour duration. The pain was retrosternal, was crushing, and was associated with profuse sweating. There was no radiation of pain, breathlessness, nausea, or vomiting. He is a known hypertensive on telmisartan. He did not have any other risk factors for coronary artery disease. He was not a smoker, and there was no family history of coronary artery disease. Initial ECG in the emergency room showed ST segment elevation in leads II, III, AVF, V5, and V6. There was reciprocal ST depression in lead AVL, and there was no right ventricular involvement or any conduction disturbances (Figure 1). On physical examination, his blood pressure was 120/80 mm of Hg and heart rate was 52/minute, and there were no abnormal findings on physical examination.

**Figure 2:**
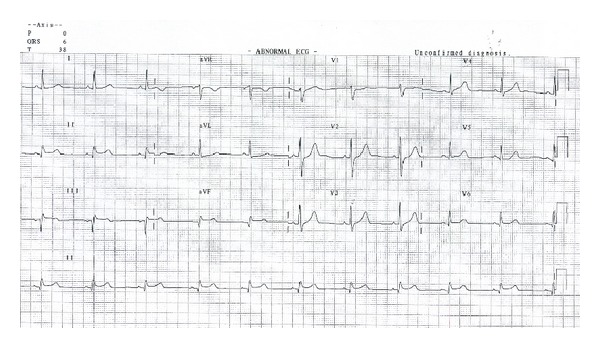
12-lead ECG after thrombolysis with tenecteplase. There is ST segment resolution with small q wave in leads II and AVF.

The patient was administered Aspirin 300 mg orally and clopidogrel 300 mg orally, and thrombolytic therapy was given with intravenous bolus tenecteplase 9000 units followed by enoxaparin 30 mg intravenously and 80 mg subcutaneously twice daily on the subsequent days. Chest pain subsided; ST segment showed resolution (Figure 2), and it was accompanied by runs of accelerated idioventricular rhythm on the cardiac monitor indicating successful thrombolysis. Cardiac enzymes showed early peaking, and the biochemical examination showed elevated total cholesterol, LDL cholesterol and triglycerides, and normal HDL cholesterol.

**Figure 3:**
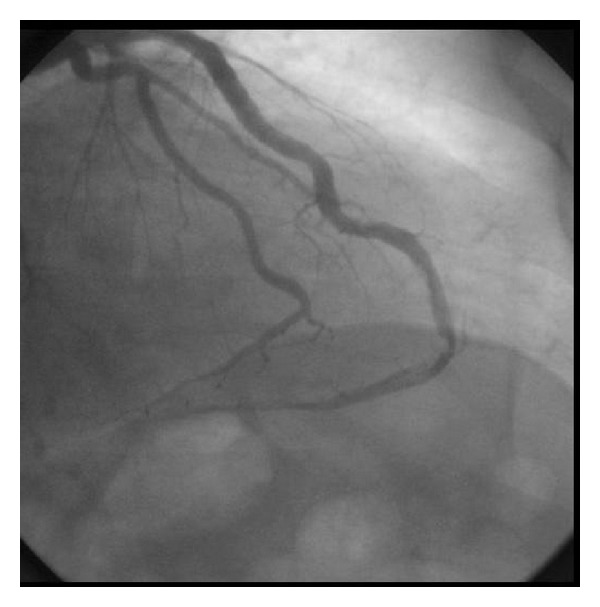
Coronary angiogram showing left anterior descending coronary artery in the right anterior oblique view. LAD is long wrapped around the apex and continues as the posterior descending artery. There is an eccentric plaque with thrombus at the apex where it continues as PDA.

He was taken up for coronary angiography after 48 hours for complete revascularization. Coronary angiography showed left dominant system. Right coronary artery was small, nondominant and ends by supplying the right ventricular branches. Left circumflex artery was large ecstatic vessel and gave rise to two large obtuse marginal branches one of which supplies the posterolateral territory. LAD was a large vessel curving around the apex of the left ventricle and continuing as the PDA, and supplying most of the inferior septum and the inferior wall. Distal LAD at the apex was showing 75% narrowing with a thrombus on it (Figure 3).

**Figure 4:**
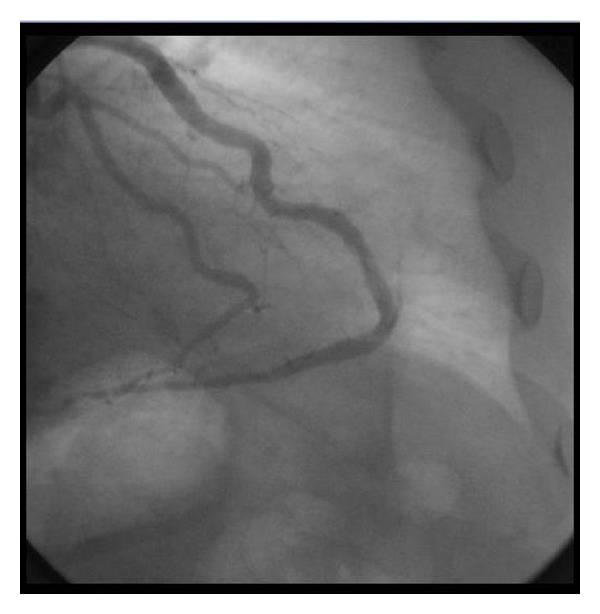
Coronary angiogram in the right anterior oblique view after deployment of the stent. Well-deployed stent is seen without any residual stenosis.

The lesion was crossed with BMW guide wire and predilated with Cross rail 2.5 × 15 mm balloons. Attempt to stent the lesion was met with difficulty in negotiating the stent through the tortuous LAD distally due to poor guiding catheter support. This was overcome by positioning another stiff guide wire in the PDA to give an extra support to guiding catheter. The guide wire was removed after positioning the stent, and the lesion was stented with Xience V (Abbott Vascular) 3 × 20 mm stent at 18 atmospheres. Post deployment angiography showed good result with TIMI III flow and no residual stenosis (Figure 4).

Patient was discharged on Aspirin 100 mg/day, clopidogrel 75 mg/day, telmisartan 80 mg/day, and pravastatin 40 mg/day. Our patient has been asymptomatic since then and is on regular followup for more than two years after procedure. 

## 3. Discussion

Acute inferior wall myocardial infarction is usually due to occlusion of the RCA and is rarely due to occlusion of LCX. Several ECG criteria have been developed to differentiate the culprit lesion in the setting of acute inferior wall myocardial infarction (MI). Simultaneous anterior and inferior myocardial infarction due to distal LAD occlusion have been described, but isolated inferior wall infarction due to LAD occlusion is rarely reported. In our case, this was due to unusual anatomy where long wrapped LAD continues to form the PDA which supplied most of the inferior wall. 

A “wrapped LAD” is defined as a LAD from a postreperfusion coronary angiogram that perfuse at least one-fourth of the inferior wall of the left ventricle in the right anterior oblique projection [2]. If the patient has a wrapped LAD and the location of the occlusion is proximal to D1, ST is elevated in anterior leads and remains isoelectric in the inferior leads. If the patient has a wrapped LAD and location of the occlusion is distal to D1, ST segment is elevated in anterior and inferior leads simultaneously [1]. Our case was an unusual variety of wrapped LADs where the entire posterior descending artery was formed by continuation of the distal LAD. The occlusion was distal to all diagonal branches and hence resulted in isolated inferior wall MI without anterior wall changes. 

Continuation of the left anterior descending coronary artery to form the posterior descending artery is rare coronary anomaly [3]. Clark et al. [4] identified a patient in whom LAD formed the PDA and terminated just before the crux. Akedemir et al. [5] described a patient with acute anterior and inferior wall myocardial infarction due to occlusion of the wrapped LAD. The patient's angiogram showed wrapped LAD with proximal and distal LAD lesions. Anterior ST segment elevation returned to isoelectric line after primary direct stenting to proximal LAD, but the inferior ST segment elevation persisted due to distal LAD lesion at the apex which could not be crossed with the guide wire.

Our case describes a rare form of left dominant coronary circulation in which LAD wraps around the apex forming posterior descending coronary artery. This has resulted in isolated inferior wall myocardial infarction due to distal LAD occlusion. Physicians and cardiac surgeons should be aware of such a variant as it has considerable impact on the clinical course due to large area of myocardium in jeopardy if the occlusion occurs in such a LAD [6].
